# Physiologically Based Pharmacokinetic Modeling of Caffeine in Preterm Neonates: Influence of Renal Function and Impairment on Dosing

**DOI:** 10.1002/jcph.70135

**Published:** 2025-11-27

**Authors:** Nolan Thomas, Matthew W. Harer, Sin Yin Lim

**Affiliations:** ^1^ Pharmacy Practice and Translational Research Division, School of Pharmacy University of Wisconsin‐Madison Madison WI USA; ^2^ Department of Pediatrics University of Wisconsin School of Medicine and Public Health Madison WI USA

**Keywords:** caffeine, neonates, PBPK, pharmacokinetics, preterm

## Abstract

Currently, the same weight‐based caffeine citrate dosing regimen is applied to all neonates. However, due to differences in growth trajectories by gestational age (GA) and altered caffeine elimination in neonates with renal injury, optimal dosing regimens may differ. In this study, we refined the existing physiologically based pharmacokinetic (PBPK) model for caffeine in preterm neonates (GA 25–32 weeks) using Simcyp to evaluate dosing across varying ages and renal function. Real‐world data were used to generate weight‐for‐age growth curves and create virtual preterm populations. CYP1A2 ontogeny model was updated to better reflect the reduced metabolic activity of caffeine in preterm neonates. A 13.7‐fold increase in glomerular filtration rate‐adjusted renal clearance was needed to match observed data. Additionally, a higher volume of distribution (0.96 L/kg) was required to account for increased body water. The final model was verified using clinical pharmacokinetic data and used to simulate plasma concentration–time profiles. Our simulations showed that more premature neonates (≤28 weeks GA) may require lower weight‐based maintenance dosing (8 mg/kg) compared with those with higher GA (10 mg/kg), and may also require an increase in doses after 4–6 weeks of therapy to maintain therapeutic levels. Neonates with significantly reduced renal function (25% of normal) may need a two‐ to threefold dose reduction. Future studies should aim to define optimal therapeutic targets, as caffeine use continues to expand.

## Introduction

Preterm birth occurs in 10% of pregnancies and is one of the leading causes of neonatal morbidity and mortality among infants in the United States and worldwide.^1^, ^2^ Due to advancements in treatment strategies, the survival rate among preterm neonates has significantly improved in the last decades.^3^ This, however, led to a higher rate of prematurity‐related complications such as apnea of prematurity and bronchopulmonary dysplasia (BPD). Caffeine, a respiratory stimulant, is the third most used medications in the neonatal intensive care unit (NICU) for the last two decades.^4^, ^5^ It was approved by the FDA in 1999 for its use for apnea of prematurity in preterm neonates with a gestational age (GA) of 28 to <33 weeks, at the dosage of 20 mg/kg loading dose followed by 5 mg/kg daily maintenance dose.^6^ In 2020, the FDA label removed the GA specification, as caffeine was found to be safe in more premature neonates—caffeine now is commonly used in preterm neonates as early as 22 weeks GA at maintenance doses of 5–10 mg/kg daily.^7^, ^8^ Currently, the same weight‐based dosing of caffeine citrate is used in preterm neonates of different ages. This dosing strategy may be suboptimal because organ function and body composition in neonates change rapidly, resulting in varying pharmacokinetics of caffeine among neonates with different prenatal maturity and postnatal ages (PNAs; postnatal maturation).^9^

Initially used for apnea of prematurity, caffeine has later been shown to decrease the need for mechanical ventilation and oxygen therapy and reduce the risk of BPD.^10^ BPD results from lung injury or disrupted lung development in premature neonates and is associated with an increased risk of death among preterm infants. Despite the common use of caffeine, BPD still affects 1 in 3 preterm neonates with GA <28 weeks.^11^ Furthermore, the rate of grade 2–3 BPD among preterm neonates of GA 26–28 weeks increased from 13% to 26% between 2012 and 2021.^12^ Evidence suggests that earlier initiation and higher doses may reduce the risk of BPD;^13^ however, the optimal dosing remains unclear, as the impact of patient‐specific factors on pharmacokinetics and the target plasma concentrations needed to prevent BPD are still unknown. More recently, caffeine has been shown to be associated with improved neurodevelopmental outcomes and reduced acute kidney injury (AKI) rates in preterm neonates.^14^, ^15^, ^16^ AKI has been independently linked to increased mortality and longer hospital stays.^17^ While understanding the pharmacokinetics/pharmacodynamics (PK/PD) of caffeine for this indication is needed to identify optimal dosing, it also highlights the need to evaluate the pharmacokinetics and renal dosing adjustments in preterm neonates who develop AKI.

In adults, caffeine is cleared rapidly with a half‐life of 5 h. It is primarily metabolized by the enzyme CYP1A2 into paraxanthine, an active metabolite, with 3% or less being excreted unchanged in the urine. In preterm neonates, however, the half‐life of caffeine is prolonged (72–96 h), with approximately 86% of caffeine being excreted unchanged in the urine due to the delayed ontogeny of CYP1A2, which develops between 1 month and 1 year of age.^7^, ^18^, ^19^, ^20^ As such, renal function in preterm neonates play a significant role in the disposition of caffeine. The impact of renal function is evident in the dosing of drugs primarily cleared by the kidneys (e.g., aminoglycosides and vancomycin), where weight‐based dosing strategies are influenced by postmenstrual age (PMA), or GA and PNA. Yet, the same weight‐based dosing of caffeine citrate is administered to preterm neonates across different ages. In this study, we aimed to refine an existing physiologically based pharmacokinetic (PBPK) model to quantify the renal and metabolic clearance of caffeine in preterm neonates across GA of 25–32 weeks, evaluate dosing regimens based on varying patient factors (age and weight), and assess the impact of renal impairment.

## Methods

### Study Design

Institutional review board approval and patient informed consent do not apply, as this study did not meet the criteria for human subjects research. We used PBPK modeling software Simcyp (version 24, SimCYP Ltd, Sheffield, UK) to perform modeling and simulations. Caffeine PBPK models in adults and children have been published previously with appropriate validation.^21^ However, extrapolation of the PBPK models to neonates did not accurately capture the mechanism of elimination in preterm neonates, as the models predicted metabolic clearance to be the primary route of elimination,^21^, ^22^ whereas renal clearance is known to predominate.^18^ Using the published caffeine PBPK model as the base model,^21^ we aimed to refine the existing neonatal PBPK model in the preterm population (GA 25–32 weeks), following a predict–learn–confirm approach. All simulations conducted during model development and verification were performed using virtual populations with demographics similar to those of the clinical study participants.^19^, ^23^, ^24^, ^25^, ^26^, ^27^, ^28^ Caffeine concentration–time data in preterm neonates from published studies were extracted using WebPlotDigitizer (http://automeris.io/WebPlotDigitizer/). In this study, caffeine concentration data refer to the concentrations of caffeine base, and caffeine dosing is based on caffeine citrate, which contains 50% caffeine base.

### Virtual Preterm Population

In the default Simcyp preterm population, the height and weight data are generated based on the UK growth charts for preterm neonates.^29^, ^30^ We compared the demographic data from this default population with the real‐world data from the Safety and Efficacy of Caffeine Citrate in Premature Infants (CAF01) study published in the NICHD Data and Specimen Hub.^8^ The study included 87,851 preterm neonates (GA <33 weeks) from 316 NICUs in the United States.^8^ The age–weight relationship was found to differ significantly. These differences may be due to the fact that preterm growth charts, such as the UK growth charts^30^ and the Fenton growth charts,^31^ do not incorporate actual postnatal growth patterns. Additionally, neonates receiving caffeine therapy may have morbidities that affect growth trajectories. Therefore, we utilized a piecewise quadratic model reported by Ehrenkranz and colleagues^32^ to update the relationship between weight (Wt) and PNA of the virtual subjects:

$$\mathrm{Wt}\;=\left\{\begin{array}{c} b+\beta_{1}\cdot PNA+\beta_{2}{\left(T_{1}-\mathrm{PNA}\right)}^{2}+\beta_{3}{\left(T_{2}-\mathrm{PNA}\right)}^{2},\;\mathrm{for}\;PNA\leq T_{1}\\ b+\beta_{1}\cdot PNA+\beta_{3}{\left(T_{2}-\mathrm{PNA}\right)}^{2},\;\mathrm{for}\;T_{1}<PNA\leq T_{2}\\ b+\beta_{1}\cdot PNA,\;for\;PNA>T_{2} \end{array}\right.$$

where b, $\beta_{1}$, $\beta_{2}$, $\beta_{3}$, $T_{1}$, and $T_{2}$ are estimated parameters based on the regression analysis. Each GA group had its own set of estimated parameters. Other physiological parameters, which are correlated with age, weight, and/or height, are derived based on the default models in the Simcyp Simulator.^29^

### Neonatal PBPK Model Development and Evaluation

The parameters used in the base model for adults and children are shown in Table 1. A minimal PBPK model was used as the drug distribution structure. The initial predicted steady‐state volume of distribution (V_ss_) was lower than observed values; therefore, it was set to 0.96 L/kg, based on reported values in preterm neonates (mean volume of distribution [V_d_] range: 0.7–1.1 L/kg).^19^, ^23^, ^24^, ^25^, ^26^, ^27^, ^28^ The coefficient of variation (CV%) was set to 30%.

**Table 1:** Input Parameters for Caffeine Base and Preterm PBPK Model

Parameter	Base Model^21^	Final Preterm Model
Physiochemical Properties		
Molecular weight (g/mol)	194.19	194.19
LogP	−0.07	−0.07
pKa	1.05 (monoprotic base)	1.05 (monoprotic base)
B/P	0.977	0.977
Fu	0.68	0.68
Plasma binding protein	Human serum albumin	Human serum albumin
Absorption		
Absorption Model	First Order Absorption Model	N/A^b^
Fa	1	N/A
Ka (1/h)	2.18	N/A
fu_gut_	1	N/A
Q_gut_ (L/h)	16.0^a^	N/A
P_eff,man_ (10^−4^ cm/s)	5.844^a^	N/A
Permeability assay, value (10^−6^ cm/s)	Caco‐1 (7.4:7.4, P&A), 30.8 (scalar: multiple; 1.91)	N/A
Distribution		
Distribution Model	Minimal PBPK	Minimal PBPK
V_ss_ (L/kg)	0.408^a^	0.96
Prediction method	Method 2	User input
Elimination		
Clearance Model	Enzyme Kinetics	Enzyme Kinetics
CYP enzyme (recombinant): V_max_ (pmol/min/pmol of isoform), k_m_ (µmol)	CYP1A2 (N1‐dm): 0.56, 157 CYP1A2 (N3‐dm): 13.6, 300 CYP1A2 (N7‐dm): 0.21, 245 CYP2E1 (N1‐dm): 0.03, 1411 CYP2E1 (N7‐dm): 0.02, 823 CYP1A2 (OH): 0.36, 265 CYP2E1 (OH): 0.18, 1019 CYP3A4 (OH): 1.8, 45080	Same as base model, but included CYP1A2 ontogeny function: $\mathrm{Ontogeny}=0.5702\times \mathrm{PM}A^{2}-0.4812\times \mathrm{PMA}+0.1165$
CL_R_ (L/h)	0.038	$C\;L_{R,\mathrm{neonate}}=CL_{R,\mathrm{adult}}\times \frac{\mathrm{GF}R_{\mathrm{neonate}}}{\mathrm{GF}R_{\mathrm{adult}}}\times 13.7$

logP, octanol–water partition coefficient; pKa, acid dissociation constant; B/P, blood to plasma binding ratio; fu, fraction unbound; Fa, fraction available from dosage form; Ka, absorption rate constant; fu_gut_, fraction unbound in enterocytes; Q_gut_, flow of gut in gut model; P_eff,man_, human jejunum effective permeability; P&A: passive and active permeability; V_ss_: volume of distribution at steady state; V_max_: maximum rate of metabolism; k_m_, Michaelis–Menten constant; dm, demethylation; OH, hydroxylation; CLR, typical renal clearance of healthy 20–30 year old male.

^a^ Predicted values from Simcyp.

^b^ Only intravenous administration was used in simulations.

A stepwise approach was used to develop the clearance model. First, the CYP1A2 ontogeny function (OF) was evaluated and updated. The OF reflects the relative enzyme abundance of CYP1A2 in preterm neonates compared with adults. The default CYP1A2 ontogeny model in Simcyp appears to overestimate the metabolic clearance of caffeine in preterm neonates, due to the inaccurate assumption that caffeine is primarily eliminated by hepatic clearance.^22^ Therefore, we used CYP1A2 enzyme activity data pooled from multiple studies in preterm neonates, as reported by van Groen et al,^33^ to create an updated OF. Using the updated model for CYP1A2, we compared the predicted metabolic clearance of neonates with a GA of 29 weeks and a PNA of 15 days with reported values.^18^, ^34^ These reported values show that metabolic clearance accounts for 14%–30% in preterm neonates with a mean GA of 29 weeks and a PNA of 6–15 days^34^ and 15% in those with a PNA of 3–21 days.^18^ We found that our CYP1A2 model overestimated caffeine metabolic clearance; however, the CYP1A2 ontogeny reported by Sonnier and Cresteil,^35^ which was one of the studies included in the pooled analysis by van Groen et al,^33^ best described the observed metabolic clearance in preterm neonates. CYP2E1 and CYP3A4 are also involved in the metabolism of caffeine. Their contributions are negligible in neonates; therefore, no further optimization was performed.

Second, we evaluated the predicted renal clearance against the observed renal clearance (70%–85% of total clearance) of neonates with a GA of 29 weeks and a PNA of 15 days. By default, the scaling of renal clearance is based on the changes in the glomerular filtration rate (GFR). We used the neonatal GFR model developed by Wu et al^36^ to estimate the GFR in preterm neonates, rather than using the default PMA‐based model. This model included birth weight, GA, and PNA as covariates, accounting for the effects of both prenatal and postnatal development on GFR. Since caffeine is primarily eliminated through renal clearance and is used in a wide span of GAs and PNAs, this GFR model provides a more accurate description of changes in renal function in preterm neonates. Particularly, it captures the varying rates of postnatal GFR maturation. The following equation was used to scale renal clearance from adults to neonates (CL_R,neonate_):

$$C\;L_{R,\mathrm{neonate}}=\;CL_{R,\mathrm{adult}}\times \frac{\mathrm{GF}R_{\mathrm{neonate}}}{\mathrm{GF}R_{\mathrm{adult}}}\times \mathrm{MF}$$

wheret CL_R,adult_ is the renal clearance in adults, and GFR_neonate_ and GFR_adult_ represent the GFR in children and neonates, respectively. A maturation factor (MF) was included to account for maturation‐related changes in renal clearance in neonates. Higher renal clearance after scaling based solely on GFR has been observed in children and neonates for drugs that are renally eliminated with significant tubular reabsorption.^37^, ^38^, ^39^ In neonates, this is likely due to immature renal function, limited urine‐concentrating ability, and increased urine flow, all of which may contribute to reduced tubular reabsorption and thereby increased renal clearance.^40^

Third, after determining the ontogeny function of CYP1A2 for metabolic clearance and the maturation factor for renal clearance in neonates with a GA of 29 weeks and a PNA of 14 days, the model was extended to other GAs and PNAs to assess the need for further modifications. The final model was verified by comparing the predicted total clearance with the reported values in the literature.

The performance of the plasma concentration prediction was considered acceptable if the predicted average pharmacokinetic parameters such as clearance and V_d_ were within twofold of the observed values. The accuracy of model predictions was evaluated by determining the average fold error (AFE) of the median predicted concentrations versus the observed concentrations based on the following equation:

$$\mathrm{AFE}\;=10^{\frac{1}{n}\sum \log\neq \left(\frac{\mathrm{predicted}}{\mathrm{observed}}\right)}$$

wheret n is the number of predicted‐observed pairs, and model acceptance criteria were defined as AFE within 0.5–2 (i.e., twofold error). The model prediction precision was evaluated by calculating the absolute average fold error (AAFE):

$$\mathrm{AAFE}\;=10^{\frac{1}{n}\sum \left|\log\neq \left(\frac{\mathrm{predicted}}{\mathrm{observed}}\right)\right|}$$

wheret the model acceptance criteria were defined as AAFE <2. The model was also evaluated based on a visual inspection of observed concentration–time data versus the predicted mean and 5th–95th percentile concentration–time data. The final neonatal PBPK model was applied to simulate the pharmacokinetic profiles of various dosing regimens among preterm neonates with different patient factors such as varying GAs, PNAs, and renal functions.

### Sensitivity Analysis

A sensitivity analysis was performed to determine the effect of GFR on caffeine concentration–time profiles due to renal clearance being the primary route of elimination of caffeine in preterm neonates. To simulate varying degrees of renal impairment, GFR was scaled in 25% increments, ranging from normal function (100%) to severe impairment (25%). Simulations were done on virtual populations of 500 preterm neonates of GA 25, 29 and 32 weeks in order to see the differences in exposure between the lowest, highest, and median GAs out of our virtual population. Mean starting age for each cohort was 1‐day PNA. Each cohort was given the same dosing regimen of intravenous caffeine citrate: 20 mg/kg for the initial loading dose and 5 mg/kg as daily maintenance doses for 14 days.

### Dose–Exposure Assessment

To evaluate caffeine exposure for different intravenous dosing regimens for preterm neonates (GA 25–32 weeks), three dosing regimens were simulated until a PMA of 34 weeks, starting on PNA day 1. Most preterm neonates do not need caffeine therapy beyond 34 weeks of PMA. Each simulated regimen is expressed as caffeine citrate dose: (1) a 20 mg/kg loading dose with 5 mg/kg maintenance doses (FDA‐recommended dosage^7^), (2) a 20 mg/kg loading dose with 10 mg/kg maintenance doses (a higher‐dose regimen used in clinical practice^41^), and (3) a 20 mg/kg loading dose with 8 mg/kg maintenance doses (the dosage used at our institution). Each dose was given as an intravenous infusion over 15 min, and each maintenance dose is given once per day, starting 24 h after the initial loading dose.

These simulations were performed on virtual populations of GA 25–32 weeks (total of 8 GA groups) using our final preterm neonatal model. Simcyp's “Redefine Subjects Over Time” function, where physiological parameters were updated every 12 h, was used to account for the rapid changes in physiologic function in neonates over time.^42^ Optimal target plasma concentrations of caffeine for the prevention of BPD or AKI are currently unknown. Caffeine concentrations above 15 mg/L have been reported to be associated with improved responses for apnea of prematurity and potentially reduced risk of BPD.^43^ The toxicity threshold is also relatively unclear, though increased risks of adverse events at plasma concentrations above 50 mg/L have been reported.^7^ The target 15–50 mg/L therapeutic window was used in our dose exposure assessment.

## Results

### Preterm Neonatal Population

**Figure 1:**
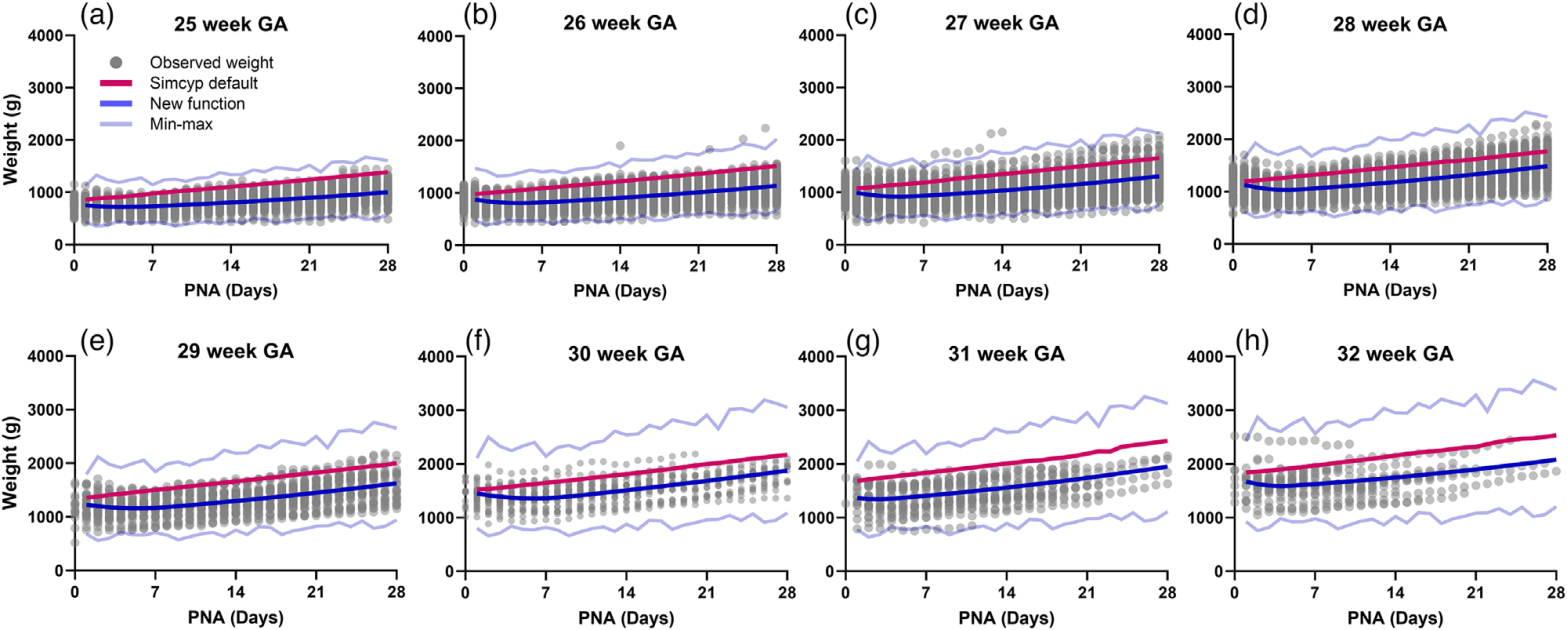
Preterm neonate populations using updated weight–age relationships based on real‐world data.

A new weight–age relationship was determined and used to develop a preterm population file (Figure 1 and Table S1). Our new weight–age relationship more accurately described the growth trajectory in preterm neonates seen in the NICU compared with the weight–age relationship used in the default Simcyp preterm population file, which tends to overpredict body weight in the simulated population.

### Preterm Neonatal PBPK Modeling

Overall, our finalized preterm PBPK model was able to accurately describe the concentration–time profiles and pharmacokinetic parameters (Table 1). When extrapolated from the adult and pediatric PBPK model, the predicted V_ss_ of caffeine was underpredicted in the preterm population compared with observed values reported in the literature.^19^, ^23^, ^24^, ^25^, ^26^, ^27^, ^28^ Assigning a V_ss_ value of 0.96 L/kg with 30% variability accounted for the higher volume of distribution observed in preterm neonates compared with other populations. The predicted V_ss_ values were 0.98‐ to 1.52‐fold of the observed values reported in various clinical pharmacokinetic studies (Table 2).

**Table 2:** Demographics and Pharmacokinetic Parameters From Published Studies Included in Our Model Development and Verification

Study	Population	Dosing Regimen and PK Model	Observed PK Parameters	
[23]	Country: Australia N: 110 Female: 53% GA: 24–29 weeks PNA: 1–45 days WT: 0.655–1.857 kg	High dose: LD: 80 mg/kg MD: 20 mg/kg/day Low dose: LD: 20 mg/kg MD: 5 mg/kg Model: 1‐compartment	$\mathrm{CL}\left(L/h\right)=0.167\cdot {\left(\frac{\mathrm{WT}}{70}\right)}^{0.75}\cdot {\left(\frac{\mathrm{PNA}}{12}\right)}^{0.358}$ IIV: 18.8%	$V_{d}\left(L\right)=58.7\cdot \left(\frac{\mathrm{WT}}{70}\right)$ IIV: 22.3%
[26]	Country: Portugal N: 75 Female: 44% GA: 23–35 weeks PNA: 1–78 days WT: 0.6–2.0 kg	LD: 17.4–21.3 mg/kg MD: 2.14–9.47 mg/kg/day Model: 1‐compartment	If GA > 28 weeks: CL(mL/h)=(5.81∗WT+1.22∗PNA)*1 If GA ≤ 28 weeks: CL(mL/h)=(5.81∗WT+1.22∗PNA)*0.757 IIV: 14.9%	$V_{d}\;\left(\mathrm{mL}\right)=911\cdot \mathrm{WT}$ IIV: N/A
[24]	Country: Australia N: 89 Female: 41% GA: 24–31 weeks PNA: 2–15 days WT: 0.57–2.3 kg	Randomly assigned: 30 mg/kg/day 15 mg/kg/day 3 mg/kg/day Model: 1‐compartment	CL(L/h)=0.00000399·WT(g)+0.000128·PNA IIV: 25%	If GA > 28 weeks: $V_{d}\left(L\right)=0.000764\cdot WT\left(g\right)+0.0468\cdot PNA$ If GA ≤ 28 weeks: $V_{d}\left(L\right)=0.000755\cdot \mathrm{WT}\left(g\right)+0.0224\cdot \mathrm{PNA}$ IIV: 11%
[25]	Country: Scotland N: 60 PMA: 25–41 weeks PNA: 1–100 days WT: 0.6–2.9 kg	LD: 20 mg/kg MD: 5 mg/kg/day Model: 1‐compartment	CL(L/d)=0.14·WT+0.0024·PNA IIV: 29%	$V_{d}\left(L\right)=0.82$ IIV: N/A
[19]	Country: Canada N: 18 GA: 25–34 weeks PNA: 3–32 days WT: 0.685–1.87 kg	LD: 10–20 mg/kg MD: 20–60 mg/kg/day	CL (mL/kg/h): Mean: 8.9 Range: 2.52–16.81	V_d_ (L/kg): Mean: 0.916 Range: 0.475–1.28
[28]	Country: Israel N: 13 Female: 54% GA: 25–34 weeks PNA: 1–42 days WT: 0.92–2.06 kg	5–20 mg/kg/day	CL (mL/kg/h): Mean 8.5 Range: 5.8–12.2 CV%: 19.1	V_d_ (L/kg): Mean: 0.78 Range: 0.47–1.01

GA, gestational age; PNA, postnatal age in days; PMA, postmenstrual age; WT, weight in kg unless noted otherwise; LD, loading dose; MD, maintenance dose; CL, clearance; V_d_, volume of distribution; IIV, inter‐individual variability; CV, coefficient of variation.

**Figure 2:**
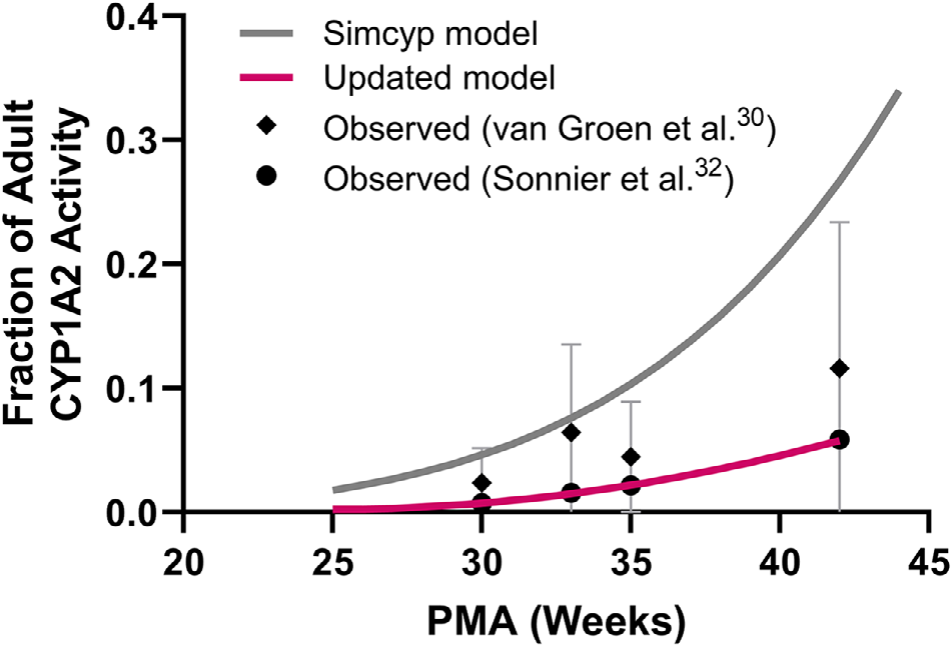
CYP1A2 ontogeny function in preterm neonates.

The default CYP1A2 ontogeny function significantly overestimated the metabolic clearance of caffeine (Figure 2). Based on the CYP1A2 metabolic activity in preterm neonates reported in Sonnier and Cresteil,^35^ we updated the CYP1A2 ontogeny function using a quadratic equation to describe its changes as a function of PMA (in years):

$$\begin{array}{ccc} \mathrm{Ontogeny}\; & = & 0.5702\times \mathrm{PM}A^{2}-0.4812\times \mathrm{PMA}\\ & & +\;0.1165 \end{array}$$

**Figure 3:**
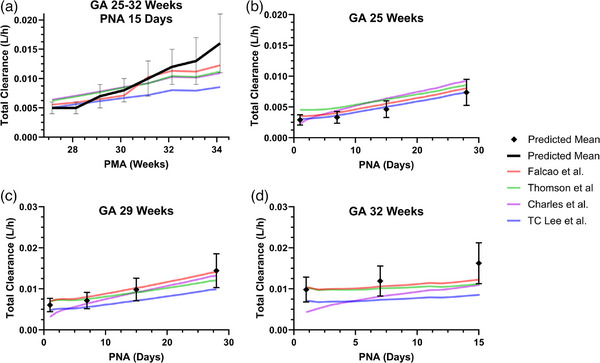
Comparison of predicted and observed total clearance across various gestational ages (GA) and postnatal ages (PNA). Postmenstrual age (PMA) is the sum of GA and PNA. Error bars represent the standard deviations.

**Figure 4:**
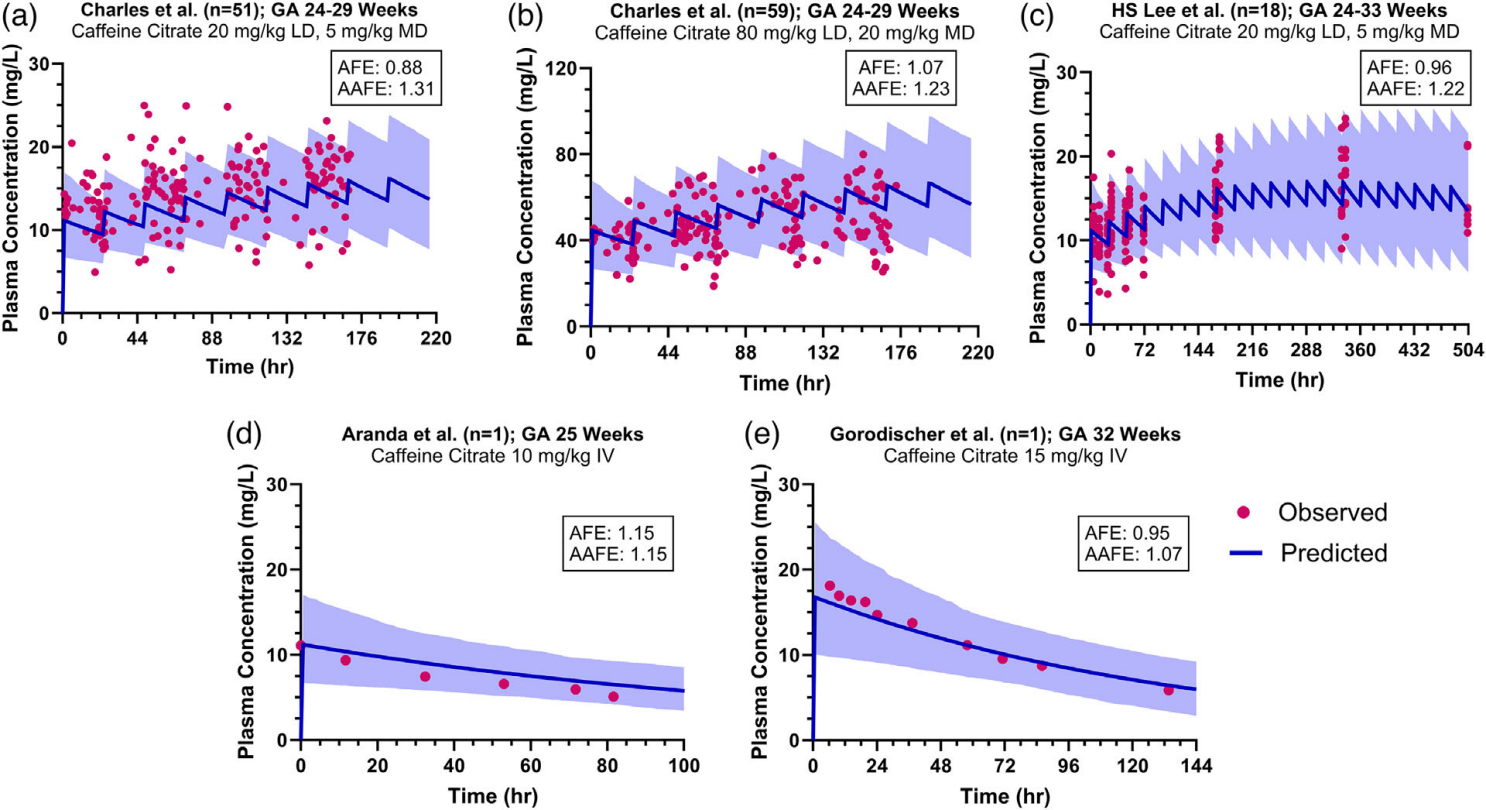
Observed and predicted plasma concentration–time profiles of intravenous (IV) caffeine in preterm neonates. Circles indicate observed concentrations; solid lines represent the predicted mean concentrations; and shaded areas denote the 5th to 95th percentile range of the predicted concentrations.

For neonates with a GA of 29 weeks and a PNA of 15 days, this model predicted metabolic clearance values that were approximately 25% of the observed total clearance, which was consistent with the current understanding of caffeine elimination pathways (15%–30% by metabolic clearance). Renal clearance, which accounts for more than 70% of the total clearance, was underestimated in preterm neonates when the PBPK model was extrapolated from children and adults based on differences in GFR. For neonates with a GA of 29 weeks and a PNA of 15 days, a maturation factor of 13.7—representing a 13.7‐fold increase in renal clearance after adjusting for differences in GFR—was needed to accurately describe the renal clearance of caffeine. The updated CYP1A2 ontogeny and renal clearance models were then expanded to other gestational ages and postnatal ages. Our predicted total clearance values at various GAs and a PNA of 15 days were 0.71‐ to 1.78‐fold of the values obtained from other published population pharmacokinetic models (Figure 3A). The predictions of total clearance across different PNAs and GAs were also adequate (Figure 3B–D). Similarly, the final PBPK model for preterm neonates successfully captured the concentration–time profiles across various dosing regimens reported in published studies, with all simulated AFE (0.88–1.15) and AAFE (1.07–1.31) values falling within our accepted range of 0.5–2 and <2, respectively (Figure 4). Relative contributions of renal clearance and hepatic CYP1A2 metabolism to the total clearance of caffeine in neonates with GA of 25, 29, and 32 weeks from birth to a PNA of 28 days are shown in Figure S1. More premature neonates have a higher fraction of renal clearance, which decreases as PNA increases.

### Sensitivity Analysis

**Figure 5:**
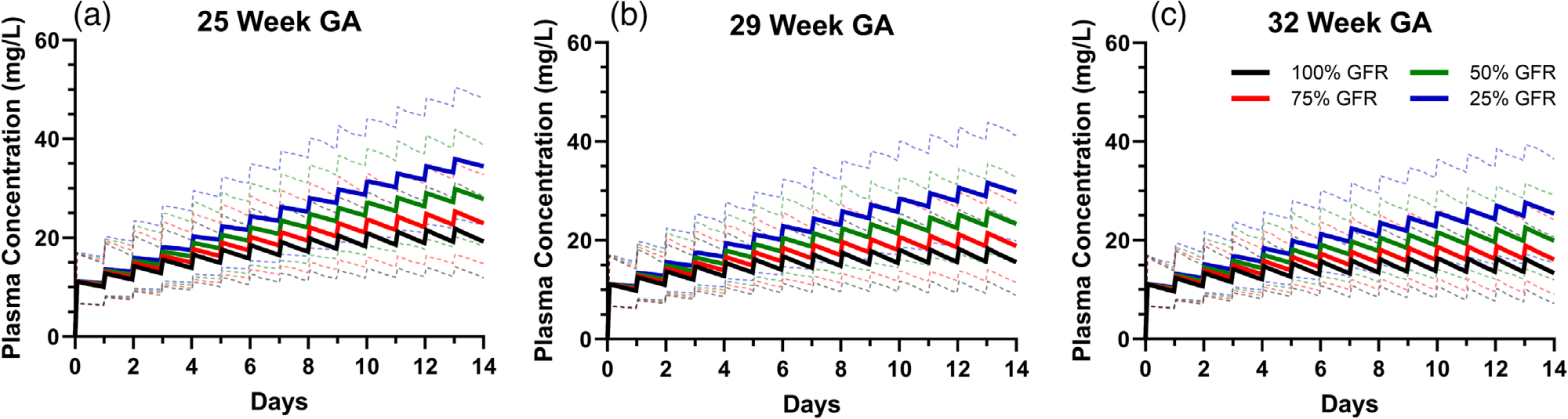
Simulated plasma concentration–time profiles of caffeine citrate (20 mg/kg loading dose followed by 5 mg/kg daily maintenance doses) in preterm neonates (GA 25, 29, and 32 weeks) with varying degrees of renal impairment, ranging from normal function (100% GFR) to severe impairment (25% GFR). Solid lines represent mean concentrations; dashed lines represent the 5th to 95th percentile range of the predicted concentrations.

Our GFR sensitivity analysis showed that reductions in GFR led to increased plasma concentrations of caffeine (Figure 5). For all three GA groups (25, 29, 32 weeks), those with only 25% of normal renal function had trough concentrations approximately twofold higher than those with normal renal function after 2 weeks of daily caffeine therapy. With longer durations of therapy (>2 weeks), this difference is expected to become more pronounced in more premature neonates (i.e., GA 25 weeks). This is because neonates with lower gestational ages and renal impairment have significantly reduced clearance and prolonged half‐life, leading to continued caffeine accumulation until steady state is reached. At a PNA of 15 days, total clearance was 3‐fold lower at 25 weeks GA, 2.5‐fold lower at 29 weeks GA, and 2‐fold lower at 32 weeks GA in neonates with 25% renal function compared with those with normal (100%) renal function.

#### Dose Exposure Assessment

**Figure 6:**
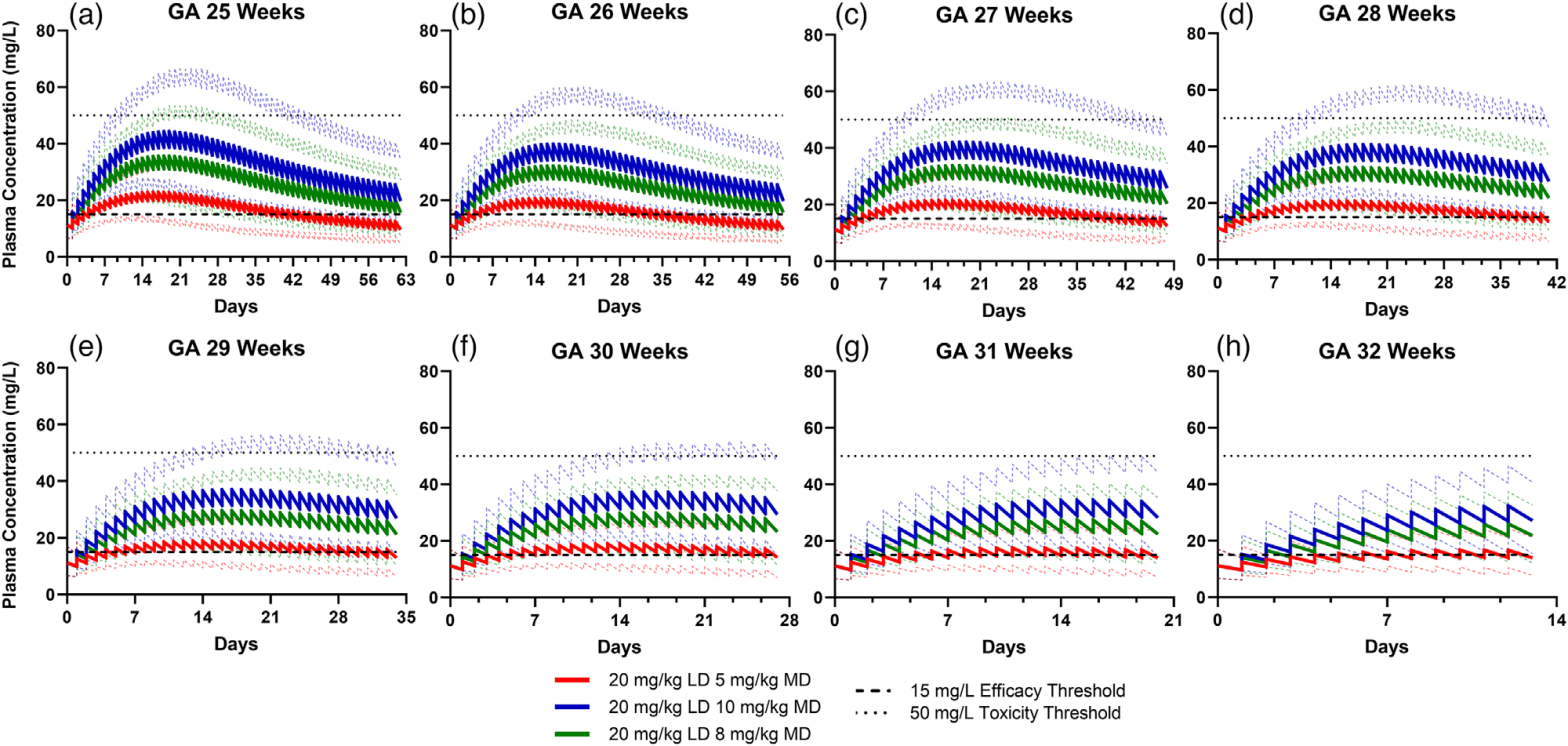
Simulated plasma concentration–time profiles of caffeine in preterm neonates given three different dosing regimens (blue, green, and red lines). Solid lines represent mean concentrations; dashed lines represent the 5th to 95th percentile range of the predicted concentrations.

Preterm neonates with GAs of 25–32 weeks and a starting PNA of 1 day were used to evaluate three dosing regimens (Figure 6). Within each regimen, plasma concentrations were higher in neonates with lower GAs. Overall, dosing regimen 1 (20 mg/kg LD, 5 mg/kg MD; FDA‐approved regimen) resulted in mean concentrations near the 15 mg/L threshold across all GAs, increasing the risk for subtherapeutic exposure. Regimen 2 (20 mg/kg LD, 10 mg/kg MD; higher‐dose regimen) was appropriate for preterm neonates *≥*29 weeks GA, with most concentrations falling within the 15–50 mg/L range. Regimen 3 (20 mg/kg LD, 8 mg/kg MD; institutional regimen) was more appropriate for preterm neonates ≤28 weeks GA, with the majority of concentrations falling within the accepted range. Among the more premature neonates (≤28 weeks GA), all regimens showed a gradual decrease in mean concentration over time, following peak concentrations around 2 weeks of therapy. This decline reflects maturation of renal and hepatic function, which increases the clearance of caffeine. To maintain caffeine concentrations within the target range of 15–50 mg/L, upward titration or an additional bolus dose may be needed after 4–6 weeks of therapy in neonates ≤28 weeks GA.

## Discussion

Neonates experience rapid physiological changes and growth in body weight and size. In preterm neonates, variations in in utero development across gestational ages significantly influence postnatal development, which may affect drug pharmacokinetics and dosing strategies. Quantifying the effects of age‐ and weight‐related factors on pharmacokinetics is challenging, as it requires large sample sizes spanning diverse age and weight ranges. This challenge highlights a limitation in population pharmacokinetic modeling. PBPK modeling, which accounts for developmental changes in target populations, can help bridge gaps in clinical research involving neonates. In this study, we refined the existing PBPK model for caffeine in preterm neonates, incorporating developmental changes in hepatic and renal clearance. Our updated CYP1A2 ontogeny better reflected the reduced metabolic activity of CYP1A2 in this population compared with the default model in Simcyp. A 13.7‐fold increase in GFR‐adjusted renal clearance was required to describe observed renal clearance in preterm neonates. Additionally, a higher volume of distribution (V_ss_ of 0.96 L/kg) was used to account for their increased body water content. Our simulations showed that greater prematurity or renal impairment may lead to significant accumulation after 2 weeks of therapy, indicating a need for dose adjustments based on GA, PNA, and renal function.

It is well‐established that caffeine has a significantly longer half‐life (3–4 days) in preterm neonates than in adults (5 h), primarily because the ontogenesis of CYP1A2 is delayed in preterm neonates.^7^, ^35^ Expression and activity of CYP1A2 are absent in fetal human liver microsomes.^35^ Within the first day postnatally, its expression remains nearly absent, rising to approximately 1.7% of adult level by 1 week, 3.5% by 1–4 weeks, and about 12% by 1–3 months of age. Since caffeine is primarily metabolized by CYP1A2, it is often used as a probe to estimate CYP1A2 activity. However, in preterm neonates, caffeine is eliminated primarily via the kidneys, with renal clearance further increased—likely due to reduced tubular reabsorption. As a result, the contribution of CYP1A2 (fm) to total clearance is significantly reduced. If altered renal clearance is not accounted for, the hepatic clearance can be overestimated, which leads to an inaccurate estimation of CYP1A2 ontogeny profiles. This may explain why the default CYP1A2 ontogeny model in Simcyp^29^, ^44^ fails to accurately predict hepatic and renal clearance of caffeine in preterm neonates, despite correctly estimating total clearance. In the default model, CYP1A2 expression in a neonate born at 28 weeks gestational age is approximately 3.2% at birth and 3.8%–6.4% at 1–4 weeks postnatally. The metabolic clearance predicted by the initial Simcyp preterm model for a 28‐week GA neonate was 0.007 L/h on day 7, 0.009 L/h on day 14, and 0.013 L/h on day 28—corresponding to 93.6%, 94.1%, and 94.8% of total clearance, respectively. These values represent overestimations of both metabolic clearance and the fraction eliminated via metabolic pathways. Our updated CYP1A2 ontogeny model, based on the metabolic activity reported by Sonnier and Cresteil,^35^ produced hepatic clearance estimates comparable to observed hepatic clearance values, which account for 15%–30% of total clearance reported in the literature.

Renal clearance of drugs undergoing extensive tubular reabsorption has been shown to be underestimated in term and preterm neonates, as well as in children, when scaled solely based on GFR.^37^, ^38^, ^39^ This may be due to reduced tubular reabsorption, which is influenced by neonates’ urine concentrating ability and increased urine flow.^40^ Additionally, tubular reabsorption matures more slowly than glomerular filtration.^45^ In adults, filtered caffeine is mostly reabsorbed (98%) by renal tubules and is not a known substrate for active secretion.^46^ These factors may collectively explain the significantly higher (13.7‐fold) renal clearance of caffeine compared with GFR‐adjusted renal clearance in preterm neonates. Given the greater contribution of renal clearance to total caffeine elimination, dose adjustments may be necessary to maintain stable therapeutic concentrations over time. We found that caffeine accumulation became more pronounced after 1–2 weeks of therapy, with concentrations reaching two‐ to threefold higher in individuals with only 25% of normal renal function. In such cases, a dose reduction of two‐ to threefold may be necessary. Interestingly, in a study evaluating the pharmacokinetics of caffeine in term neonates with hypoxic‐ischemic encephalopathy receiving cooling, the clearance (6.4 mL/h/kg) was comparable to those reported in preterm neonates (Table 2).^47^ This likely reflects their reduced renal function due to hypoxic‐ischemic injury, rather than the effect of cooling, which was not identified as a significant covariate in their model.

However, accurately assessing renal function in preterm neonates is challenging. Serum creatinine, the most commonly used biomarker, is highly variable in this population, influenced by maternal levels, and changes with time.^48^ Its normal temporary postnatal upward trends may be misinterpreted as AKI.^49^ Urine output, another criterion for AKI, is also difficult to measure reliably in neonates. Alternative urinary biomarkers such as cystatin C, NGAL, and KIM‐1 are emerging markers of renal injury. However, these lack standardization and consistent validation.^50^ Additionally, near‐infrared spectroscopy has been explored as a non‐invasive tool to assess renal oxygenation and impairment, though its routine clinical utility remains under evaluation.^51^

Caffeine citrate, given as a 20 mg/kg loading dose followed by a 5 mg/kg maintenance dose, is FDA‐approved for the treatment of apnea of prematurity in preterm neonates.^7^ However, its use has evolved to: (1) be initiated earlier to prevent apnea and potentially improve other clinical outcomes such as BPD and retinopathy of prematurity; (2) be given at higher doses to further improve such outcomes; and (3) be continued beyond the typical discontinuation age of 34 weeks' PMA to reduce the need for respiratory support and facilitate earlier hospital discharge.^52^ Furthermore, emerging evidence showed that caffeine may protect against AKI among preterm neonates. An observational study has shown that caffeine use is associated with a reduced incidence of AKI in preterm neonates (adjusted odds ratio: 0.2; 95% confidence interval: 0.11–0.34).^16^ Due to limited and sometimes conflicting evidence, questions regarding the safety, efficacy, and long‐term clinical outcomes of these evolving caffeine use practices remain unanswered. Using our final PBPK model, we showed that patient‐specific factors including GA, PNA, and renal function can have significant impact on caffeine pharmacokinetics and plasma concentrations. Specifically, when targeting a therapeutic range of 15–50 mg/L, more premature neonates (≤28 weeks' GA) may need a lower weight‐based dosing (20 mg/kg loading dose, 8 mg/kg maintenance dose) compared with those >28 weeks' GA (20 mg/kg loading dose, 10 mg/kg maintenance dose). Additionally, dose increase may be needed after 4–6 weeks of therapy among neonates ≤28 weeks' GA. While our PBPK model offers insights into pharmacokinetic variability among preterm neonates, a major question remains: what is the optimal therapeutic target range for different indications?

This study had limitations. First, our PBPK model had several assumptions. The model was developed in a stepwise manner, initially using renal elimination data from preterm neonates with a mean GA of 29 weeks. Therefore, both renal and hepatic clearance for neonates at 29 weeks' GA were verified using clinical data. However, model verification for other GA groups relied on total clearance data. We assumed that differences in GFR among preterm neonates could predict the renal clearance across different GA groups. Together with the updated CYP1A2 ontogeny model, our final model resulted in total clearance values that aligned well with those reported in the literature. Additionally, we used a single scalar (MF) of 13.7 to account for increased renal clearance due to reduced tubular reabsorption. This value is based on caffeine data; it may not fully capture the underlying mechanisms. Future work should aim to quantify the intertwining relationships between drug‐specific tubular reabsorption, its physicochemical properties, and age‐related renal maturation, so that this renal clearance model can be applied to other drugs with significant tubular reabsorption. We also assumed minimal variation in V_ss_ across different GAs, based on literature reports indicating consistent V_ss_ values, with body weight being the primary covariate. Second, our CYP1A2 maturation model is driven by PMA, and the renal function model is based on birth weight, GA, and PNA. However, other factors—such as genetic polymorphisms, pathological conditions, and concomitant medications (particularly nephrotoxic agents)—may contribute to variability in caffeine pharmacokinetics and were not accounted for in this model.^53^ This CYP1A2 model may be further tested and expanded to include other relevant drugs. Third, due to the lack of clinical data, the impact of renal impairment observed in our sensitivity analysis requires confirmation in future studies. Lastly, we only performed simulations using the intravenous route of administration. Since the bioavailability of caffeine is close to 100%, we expect the concentration trends observed in this study to be similar to those seen with oral caffeine regimens.

## Conclusions

A neonatal PBPK model for caffeine was successfully refined to determine the dose–exposure relationship in preterm neonates across a wide range of gestational ages. While several population pharmacokinetic models have identified the impact of weight and age on caffeine pharmacokinetics, our PBPK model further illustrates the impact of renal impairment on caffeine dosing. Due to differences in pharmacokinetics, more premature neonates (≤28 weeks GA) may require lower weight‐based dosing compared with those with higher GA, and may also require an increase in doses of caffeine citrate after 4–6 weeks of therapy to maintain therapeutic levels. Additionally, neonates with significantly reduced renal function (25% of normal) may need two‐ to threefold dose reduction, especially after 2 weeks of therapy when drug accumulation becomes more pronounced. Future studies should aim to define optimal therapeutic targets of caffeine, especially as its clinical use continues to expand beyond apnea of prematurity to other indications. In particular, for neonates with AKI, where caffeine may offer renal protective effects but its clearance is significantly reduced, it is crucial to establish appropriate dose adjustments. These efforts will be essential for guiding precision dosing and improving outcomes of preterm neonates.

## Conflicts of Interest

MWH and SYL receive funding from NIH NICHD (R01 1R01HD116793‐01). MWH holds Invention Disclosure #109831, P240274; and MWH is a board member of Neonatal Kidney Collaborative Board Member.

## Funding

The authors have no specific funding to disclose.

## Supporting information



Supporting Information

## Data Availability

The data are all from the published articles cited in the references.
