# Supplementary material for: Physiologically Based Pharmacokinetic Modeling of Caffeine in Preterm Neonates: Influence of Renal Function and Impairment on Dosing
Source: J Clin Pharmacol. 2025 Nov 27;66(1):e70135. doi: 10.1002/jcph.70135 (PMC12715538; doi:10.1002/jcph.70135)
Supplement: Supplementary file 1 — Supporting Information [file JCPH-66-0-s001.docx]

Supplementary Material

**Title**: Physiologically Based Pharmacokinetic Modeling of Caffeine in Preterm Neonates: Influence of Renal Function and Impairment on Dosing

**Author**: Nolan Thomas, BS^1^; Matthew W. Harer, MD^2^; Sin Yin Lim, PharmD, MS^1*^

**Affiliations**:

^1^Pharmacy Practice and Translational Research Division, School of Pharmacy, University of Wisconsin-Madison

^2^Department of Pediatrics, University of Wisconsin School of Medicine and Public Health

***Corresponding author**:

Sin Yin Lim

777 Highland Ave

Madison, WI 53705

Sinyin.lim@wisc.edu

**Table S1:** Weight-age relationship^a^ in preterm neonates.

| **GA (weeks)** | **b** | **β_1_** | **β_2_** | **β_3_** | **T_1_** | **T_2_** |
| --- | --- | --- | --- | --- | --- | --- |
| 25 | -71.60 | 29.85 | 2.46 | 0.19 | 6.05 | 63.75 |
| 26 | 77.66 | 31.52 | 3.83 | 0.19 | 5.42 | 60.10 |
| 27 | 364.78 | 31.78 | 4.56 | 0.24 | 5.20 | 45.53 |
| 28 | 593.49 | 31.94 | 6.56 | 0.27 | 4.78 | 38.01 |
| 29 | 256.41 | 41.92 | 3.22 | 0.24 | 6.97 | 58.90 |
| 30 | 280.69 | 43.88 | 3.70 | 0.15 | 7.85 | 79.33 |
| 31 | 217.36 | 51.07 | 8.32 | 0.30 | 3.81 | 59.92 |
| 32 | 290.57 | 50.28 | 9.07 | 0.29 | 3.77 | 65.37 |

^a^The equations developed by Ehrenkranz et al.^1^:

$$Wt=\left\{ \begin{aligned} b+\beta_{1}\cdot PNA+\beta_{2}\left( T_{1}-PNA \right)^{2}+\beta_{3}\left( T_{2}-PNA \right)^{2}, for PNA\leq T_{1} \\ b+\beta_{1}\cdot PNA+\beta_{3}\left( T_{2}-PNA \right)^{2}, for T_{1}<PNA\leq T_{2} \\ b+\beta_{1}\cdot PNA, for PNA>T_{2} \end{aligned} \right.$$

**
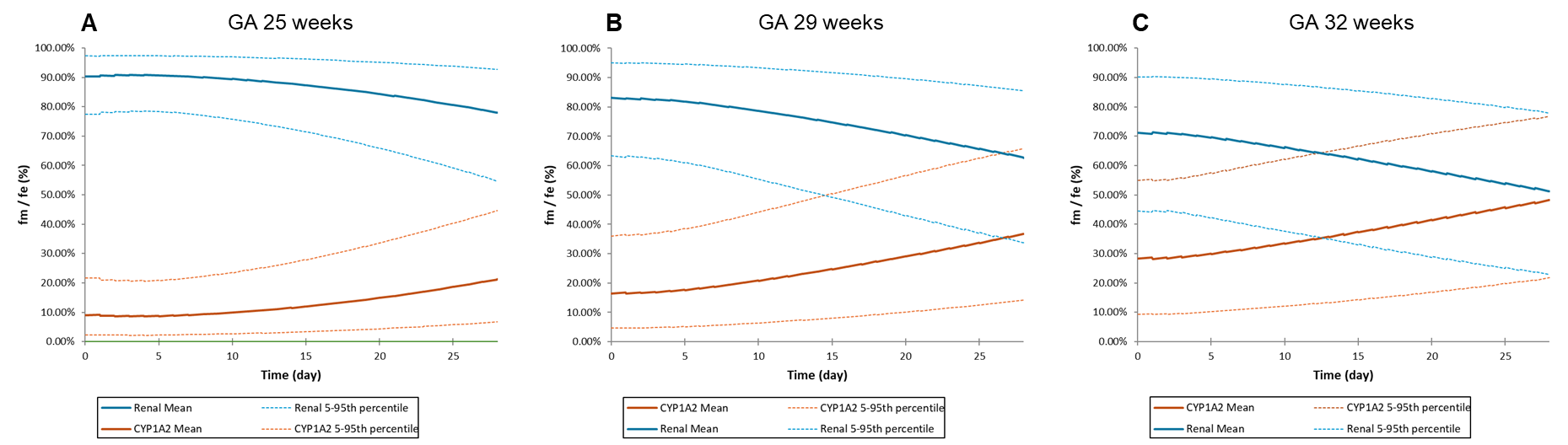
**

**Figure S1.** Relative contributions of renal clearance (fe) and hepatic CYP1A2 metabolism (fm) to the total clearance of caffeine in neonates with gestational ages of 25 (A), 29 (B), and 32 weeks (C) from birth to a postnatal age of 28 days.

**Reference:**

1. Ehrenkranz RA, Younes N, Lemons JA, et al. Longitudinal growth of hospitalized very low birth weight infants. Pediatrics. 1999;104(2 Pt 1): 280-289.
